# AAUConvNeXt: Enhancing Crop Lodging Segmentation with Optimized Deep Learning Architectures

**DOI:** 10.34133/plantphenomics.0182

**Published:** 2024-04-25

**Authors:** Panli Zhang, Longhui Niu, Mengchen Cai, Hongxu Chen, Xiaobo Sun

**Affiliations:** College of Engineering, Northeast Agricultural University, Harbin 150030, China.

## Abstract

Rice lodging, a phenomenon precipitated by environmental factors or crop characteristics, presents a substantial challenge in agricultural production, notably impacting yield prediction and disaster assessment. Despite that the application of conventional methodologies like visual assessment, mathematical models, and satellite remote sensing technologies has been employed in the segmentation of crop lodging, these approaches are still constrained in precision, immediacy, and capacity for large-scale evaluation. This study introduces an innovative convolutional neural network architecture, AFOA + APOM + UConvNeXt, that integrates intelligent optimization algorithms for automatic selection of optimal network parameters, thereby enhancing the accuracy and efficiency of crop lodging segmentation. The proposed model, empirically validated, outperforms recent state-of-the-art models in crop lodging segmentation, demonstrating higher accuracy, lower computational resource requirements, and greater efficiency, thereby markedly reducing the cost of segmentation. In addition, we investigated the segmentation on half lodging rice, and the results indicate that the model exhibits commendable performance on the half lodging dataset. This outcome holds significant implications for the prediction of rice lodging trends. The fusion of deep learning with intelligent optimization algorithms in this study offers a new effective tool for crop lodging monitoring in agricultural production, providing strong technical support for accurate crop phenotypic information extraction, and is expected to play a significant role in agricultural production practices.

## Introduction

Crop lodging is a common phenomenon in agricultural production. Lodging refers to the inability of crops to stand upright due to various external factors such as wind, rain, pest infestation, or improper agricultural management, or due to genetic or trait expressions of the crops themselves, leading to lean or fall over in the field [1]. This phenomenon occurs in many crops, including wheat, rice, corn, and barley. The impact of crop lodging on agricultural production is multifaceted [2]. On the one hand, lodging directly affects crop yield. Lodging during the crop growth stage causes overlapping of leaves, increases crop density, and reduces the leaf area exposed to sunlight, thereby affecting the efficiency of photosynthesis [3]. Additionally, lodging causes the leaves and fruits of crops to come into contact with the ground, increasing the risk of pest infestation. Lodging during the harvest stage significantly increases the difficulty of harvesting [4], especially for grains, where lodging can lead to a substantial increase in grain loss. Therefore, harvesting must be done carefully, which in turn increases labor and time costs to some extent. The causes of crop lodging in agricultural production are diverse. Among them, climatic factors are one of the main reasons. Strong winds, heavy rains, and hail can all lead to crop lodging. Moreover, soil conditions, fertilization and irrigation management, pest infestation, and improper operation of agricultural machinery can also cause lodging [5]. To prevent and reduce crop lodging, farmers and agricultural researchers have taken various measures, such as selecting lodging-resistant varieties, applying fertilizers and irrigation reasonably, timely pest control, and proper use of agricultural machinery. However, these measures cannot completely avoid the occurrence of lodging. Therefore, monitoring and predicting crop lodging situations is of great significance. Timely acquisition of crop lodging information can guide farmers or agricultural machinery operators to carry out targeted remedial measures, such as using appropriate fertilization, irrigation, and operational conditions to reduce the adverse impact of lodging on agricultural production. Moreover, timely acquisition of lodging information provides valuable data for agricultural researchers, helping them better understand the causes and mechanisms of crop lodging, and thereby develop more effective prevention and management measures.

Traditional methodologies for acquiring information on crop lodging are characterized by their labor-intensive nature, inefficiency, and poor timeliness, severely constrained by geographical, meteorological, and various external factors. These limitations render them inadequate for meeting practical demands. With the advancement of remote sensing technology, the application of this technology for monitoring crop lodging conditions has emerged as an efficient and effective alternative to conventional manual methods. In the field of satellite remote sensing monitoring, Guo et al*.* [6] explored the practicality of Sentinel-1 and Sentinel-2 data in the identification of crop lodging, achieving an overall accuracy rate of 78% for the automatic recognition of crop lodging. However, the recognition accuracy still needs to be improved. Qu et al*.* [7] conducted regional-scale monitoring of the severity of corn lodging based on time-weighted dynamic time warping of multi-temporal Sentinel-1 images, successfully categorizing the severity of corn lodging. However, the segmentation performance for local features of crop lodging is poor, mainly because crop lodging sometimes occurs only in local areas, and the low resolution of satellite remote sensing greatly limits the effective capture of local lodging areas. Although satellite data offer the advantages of covering large geographical areas and providing repeated observations, the observational scale of satellite remote sensing data is large, and its spatial resolution is relatively low, making it challenging to quantify the degree of lodging. Additionally, the timeliness of satellite observations is poor, and they are susceptible to interference from atmospheric cloud cover, often only accurately identifying lodging after it has occurred, thus introducing a certain degree of latency. Synthetic aperture radar (SAR) is an active remote sensing technology that uses radar waves (usually microwaves) to detect surface characteristics. In the field of agriculture, particularly in monitoring crop lodging, SAR has shown its potential application value. Shu et al*.* [8] conducted monitoring of corn lodging based on multi-temporal SAR data. By analyzing the optimal sensitive polarization combination of corn plant height before and after lodging, they achieved an overall accuracy rate of 67% in grading the severity of corn lodging. SAR monitoring of crop lodging has its unique advantages, such as the ability to observe under all-weather conditions, but the radar scattering characteristics of crops are affected by various factors, including the type of crop, growth stage, soil moisture, and the structural characteristics of the crops themselves. These variables increase the complexity of accurately monitoring crop lodging using SAR data. While satellite remote sensing methods are effective, their effectiveness in monitoring lodging in small-sized crops like wheat and rice still requires improvement. Due to limitations in temporal and spatial resolution, satellite remote sensing technology cannot acquire lodging information in real time and with accuracy. Furthermore, the acquisition of satellite images is easily affected by cloud cover, with long access cycles and high costs. In recent years, significant progress has been made in the monitoring and prediction of crop lodging with the development of unmanned aerial vehicle (UAV) remote sensing technology and information technology. UAVs can provide high-resolution, high-timeliness image data, allowing researchers to monitor large agricultural areas in real time, enabling farmers and researchers to assess crop conditions more accurately and detect crop lodging promptly. Shu et al*.* [9] from China Agricultural University have used UAV-based multi-temporal digital images to assess the severity of corn lodging. They employed two linear regression and three machine learning methods to classify maize lodging severity and used a random forest model to map the severity of corn lodging in the study area. Although the random forest model may perform well within the study area, its generalizability (its performance under different areas and environmental conditions) remains unknown. The soil types, climate conditions, and crop varieties of different geographic locations may affect the accuracy and reliability of the model. Zhang et al*.* [10] utilized UAV RGB imagery to improve the detection performance of lodging areas in wheat fields. They introduced three evaluation indicators—accuracy, potential, and stability—and selected three feature adaptive models to fully assess the lodging situation of wheat crops. For the evaluation indicators introduced (accuracy, potential, and stability), although helpful in fully assessing model performance, their accurate measurement and interpretation may be relatively complex, requiring detailed statistical analysis and interpretation, greatly limiting the widespread application of this method. Li et al*.* [11] from the Chinese Academy of Sciences proposed a UAV-based framework for effectively assessing crop lodging, concluding that UAV visible light imagery is more practical for field-scale assessment of crop lodging. However, crop lodging is not a binary state, and it is a continuous changing process, with various degrees from slight to complete lodging. This study has certain limitations in identifying these subtle differences in continuous change. Additionally, by analyzing remote sensing data, researchers can also predict the risk of crop lodging, providing decision support for farmers. Thus, based on the flexibility and timeliness of UAV platforms, the monitoring effectiveness for crop lodging shows significant advantages. However, the studies mentioned above largely rely on traditional approaches or machine learning for analyzing UAV remote sensing data, with limitations in flexibility, efficiency, and accurately pinpointing the locations of crop lodging.

In recent years, with the rapid development of artificial intelligence and deep learning, convolutional neural networks (CNN) have shown great potential in extracting information about crop lodging. Azizi et al*.* [12] developed an approach based on deep learning to analyze aerial RGB images for wheat lodging identification and classification. They employed CNN models such as ResNet50 and EfficientNet-B7 to classify wheat lodging at individual and over time, achieving improved extraction effectiveness with their refined models. Modi et al*.* [13] evaluated seven state-of-the-art (SOTA) deep learning models for the identification of sugarcane lodging. They found that the residual blocks and skip connection features of ResNet50 provided the highest accuracy (98.5%) and the lowest error rate (1%). Wang and Xiao [14] proposed a grid-level segmentation model tailored for crop lodging scenarios, combining dense connections and inception to build a feature extraction network. This approach offered a good balance between parameter efficiency, computational efficiency, and acceptable accuracy. Song et al*.* [15] conducted research on identifying the lodging status of sunflowers using UAV remote sensing imaging. They found that deep learning CNNs outperformed classical support vector machines in terms of accuracy for lodging identification. Additionally, within semantic segmentation models, an improved version of SegNet achieved the best performance in terms of accuracy and generalization. However, the current practice for determining the number of convolutional kernels and other parameters in CNNs still relies heavily on human experience or extensive repeated experiments. Researchers try different parameter combinations to see which performs best on the validation set. Therefore, the parameter choices in many of today’s popular CNN architectures are based on extensive experimental findings. This method can consume significant computational resources and time [16]. With the development of intelligent optimization algorithms, such as genetic algorithms, particle swarm optimization, and Bayesian optimization, a new approach has emerged: using these algorithms to automate and optimize the structure and parameter selection of CNNs. These algorithms can explore more optimal network configurations in a shorter time by simulating natural selection or other optimization mechanisms. For instance, genetic algorithms optimize network structures by simulating biological evolution processes like crossover and mutation [17]. Particle swarm optimization, on the other hand, finds optimal solutions by mimicking the social behavior of bird flocks or fish schools [18]. This research further explores the application of this technology in the acquisition of crop phenotypic information, potentially leading to more efficient and effective tools for tasks such as crop lodging segmentation and other agricultural monitoring needs.

Therefore, the purpose of our research is to apply intelligent optimization algorithms to optimize deep learning networks, specifically for the task of crop lodging segmentation in the agricultural field. We propose an innovative CNN architecture that integrates intelligent optimization algorithms for the automatic selection of optimal network parameters. This is aimed at automating the choice of the best network parameters to enhance the accuracy and efficiency of crop lodging segmentation. Through comparative experiments, we demonstrate the superiority of the optimized network in crop lodging segmentation tasks compared to traditional methods, including higher accuracy and lower computational resource requirements. This research not only provides a new perspective for parameter optimization in deep learning for specific applications but also offers an effective technical means for crop phenotypic information extraction.

## Materials and Methods

### Study area

**Fig. 1.**
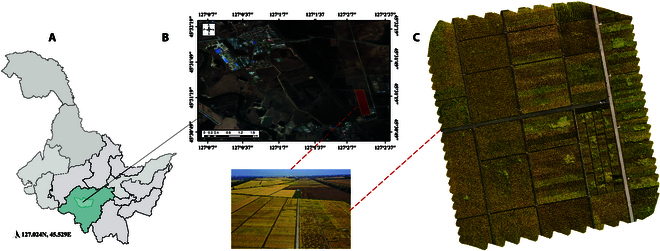
The geographic location of the experimental site. (A) Harbin City, Acheng District. (B) Scientific Research Base. (C) Imagery acquisition area.

The study area for the research is situated at the rice experimental base of Northeast Agricultural University, located in Acheng District, Harbin City, Heilongjiang Province, China. The geographic location of the experimental site is shown in Fig. 1. This site lies in a region characterized by a continental climate influenced by the Asian monsoon, with distinct seasons including freezing, dry winters and hot, rainy summers [19]. The warm season extends from around May 9 to September 21, with average daily high temperatures exceeding 19 °C, and the hottest month being July with an average high of 27 °C and a low of 16 °C. Conversely, the cold season spans from November 27 to February 22, during which the average daily high temperature falls below −6 °C [20]. The soil at the experimental base is black soil, as is common in the Harbin area. Black soil in this region is known for their high organic carbon content, with a dark to black soil surface extending to a depth of at least 25 cm. These soils are characterized by high-quality humus, resulting from a high base saturation of over 50%, and a stable aggregate structure [21]. The soil properties in Harbin, as indicated by a study, showed a soil organic matter of 26.7 g/kg and total nitrogen of a certain value, indicating a fertile ground that supports metabolic activity and microbial communities, crucial for rice cultivation [22]. The rice variety selected for cultivation in this study is the DF159 rice variety, which exhibits a suite of agronomic traits that are delineated by its growth and morphological characteristics. The developmental timeline from germination to maturity spans approximately 128 days, necessitating an accumulated temperature of around 2,350 °C at or above a base temperature of 10 °C. This cultivar typically develops 11 leaves on the main stem, manifesting an elliptic grain shape. The plant architecture is characterized by a stature reaching approximately 90 cm in height, and bearing panicles with a length of around 20 cm. Each panicle comprises about 150 grains, culminating in a thousand-grain weight of 27.4 g. The climate and soil conditions are conducive for rice cultivation, providing a suitable environment for the growth and development of the DF159 rice variety. The well-distributed rainfall during the summer months is beneficial for the rice crop, ensuring adequate water supply during the critical growth stages. Moreover, the fertile black soil provides essential nutrients and a suitable structure for rice root development, which are critical for achieving optimal growth and yield [23]. The detailed characterization of the study area including the climatic and soil conditions provides a robust foundation for understanding the environmental factors influencing the growth and performance of the DF159 in the experimental plots situated at the Northeast Agricultural University rice experimental base.

### Dataset

#### UAV platform and data acquisition

Our study employed a DJI Matrice 300 RTK UAV platform (SZ DJI Technology Co., Shenzhen, China) equipped with a DJI P1 camera with dimensions of 198 × 166 × 129 mm for capturing high-resolution imagery of the rice fields to assess lodging. The UAV was launched with a takeoff speed of 10.8 m/s and cruised over the trial area at a speed of 1.5 m/s at an altitude of 20 m, which provided a ground sample distance (GSD) of 0.55 cm/pixel. The flight path covered a length of 642 m, encompassing 13 waypoints and generating 173 images over an area of 11.1 mu (0.74 hectares). The flight configuration ensured an 80% overlap along both the flight direction and across adjacent flight lines, enhancing the spatial continuity and redundancy of the acquired imagery, which is crucial for accurate mosaicking and analysis. The data acquisition process was carried out under clear and cloudless weather conditions, minimizing atmospheric interference and ensuring consistent illumination across the study area. The UAV traversed the trial area in a fully automated flight mode, which was configured using the DJI GS Pro software. The built-in real-time kinematic (RTK) of the UAV facilitated the acquisition of high-precision position and orientation system (POS) information during image capture, which was crucial for accurate georeferencing of the data. The high-resolution imagery captured by the DJI Zenmuse P1 camera, coupled with the precise geolocation data acquired through the M300 RTK’s built-in RTK, provided a robust dataset for assessing the extent of rice lodging in the study area. Additionally, the data acquisition period spanned from September 1 to October 20, during which the rice fields underwent several stages of lodging, ranging from mild to severe. As the maturity of rice plants increases, some transition from an upright position, denoted as no lodging (NL), gradually bending into a half lodging state, referred to as half lodging (HL). Ultimately, influenced by factors such as decreased moisture content and climatic conditions, some plants reach a fully lodging state (L). To facilitate more precise segmentation of lodging in rice, this study proposes the differentiation of crop states into three categories: NL, HL, and L. The meticulous planning and execution of the UAV flights, along with the high-performance specifications of the UAV and camera, ensured the acquisition of high-quality data, setting a solid foundation for the subsequent analysis and findings of this study.

**Fig. 2.**
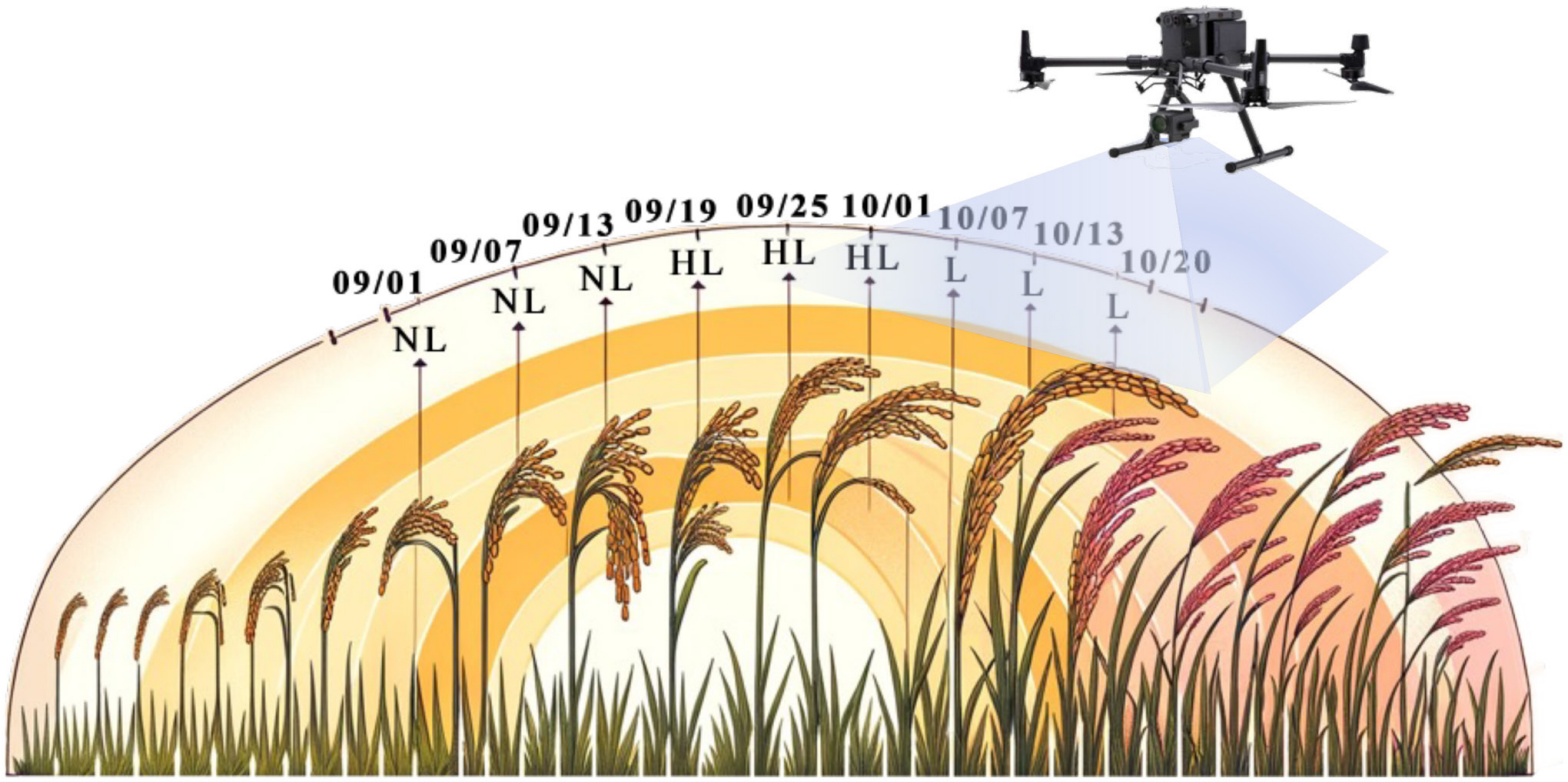
The acquisition of rice lodging information via unmanned aerial vehicles.

We utilized the UAV to continuously capture orthophotos of rice during the harvest season (from September 1 to October 20) on the temporal dimension and to persistently acquire changes in crop lodging characteristics (from NL to L) on the spatial dimension, as shown in Fig. 2. Given that the process of rice lodging unfolds gradually—transitioning from an upright state through an HL phase to full lodging—the experimental design included a sampling interval of every 6 days. This approach ensures that the collected data comprehensively span the entire rice harvest period in the temporal dimension and encapsulate the entire sequence of rice lodging phases, from NL, through HL, to L, in the spatial dimension.

#### Dataset construction

Ensuring the quality of data annotation is paramount for guaranteeing the accuracy of model segmentation. To achieve consistency in the annotation of different categories within the images, we utilized DJI Terra (SZ DJI Technology Co., Shenzhen, China), an orthomosaic software, for stitching together 173 images collected by drones. This process yielded eight digital orthophotos, which were subsequently annotated using Labelme. The categories annotated include background (BG), NL, HL, and L. Examples of the digital orthophotos and their corresponding annotations are presented in Fig. S1.

Due to the large size of the digital orthophotos, direct input into semantic segmentation models results in insufficient computer memory and GPU (graphics processing unit) memory. To address this issue, the study involved segmenting both the digital orthophotos and the manually annotated images. This segmentation aimed to preserve as much image detail as possible, enabling the model to learn image features at various scales and enhance its adaptability to scale transformations, thereby improving the model’s generalization capabilities. The digital orthophotos were segmented into sub-images with resolutions of 2,048 × 2,048, 1,024 × 1,024, and 512 × 512 pixels. Subsequently, images with resolutions of 2,048 × 2,048 and 1,024 × 1,024 were scaled down to a uniform resolution of 512 × 512 pixels, resulting in a total of 3,941 image samples with a resolution of 512 × 512 pixels, which constituted the rice lodging dataset. Examples of this dataset are shown in Fig. S2.

### UConvNeXt segmentation model

**Fig. 3.**
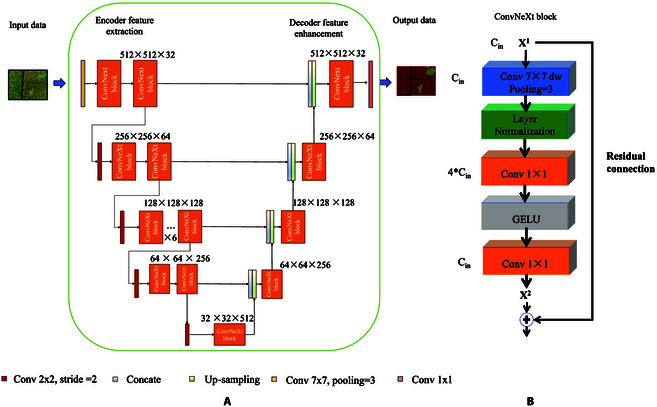
(A and B) The U-Net architecture with ConvNeXt blocks.

The Unet-ConvNeXt (UConvNeXt) segmentation model represents a novel fusion of the U-Net architecture with ConvNeXt convolutional strategies. This integration aims to leverage the strengths of both architectures to achieve high-precision semantic segmentation. The U-Net model initially designed for biomedical image segmentation stands out for its encoder-decoder structure with skip connections, enabling efficient multi-scale feature fusion [24]. This architecture is particularly advantageous in preserving detailed spatial information and context, crucial for intricate tasks like crop lodging segmentation. Compared to other segmentation models like FCN [25] or DeepLab [26], U-Net’s ability to integrate features at different scales and its robustness to variations in object size make it an ideal choice for accurately delineating the irregular and varied patterns of crop lodging. As a recent innovation in CNNs, the ConvNeXt introduces modifications to traditional CNNs by incorporating elements inspired by Transformers [27]. Its design is tailored to extract more complex and hierarchical features from images, crucial for distinguishing subtle differences in crop conditions. By replacing the conventional convolutional layers in U-Net with ConvNeXt blocks, the UConvNeXt model gains enhanced feature extraction capabilities, crucial for identifying varying degrees of lodging in dense and complex agricultural scenes. The specific structure of the UConvNeXt model is shown in Fig. 3.

In Fig. 3A, the ConvNeXt block is highlighted within a red frame, and its structure is depicted in Fig. 3B. The ConvNeXt block comprises depthwise separable convolutions that utilize 7 × 7 depthwise convolutions and 1 × 1 pointwise convolutions. The 7 × 7 depthwise convolutions, applied independently on each input channel, not only significantly reduce the computational load and model size but also extract more extensive global lodging semantic information within each channel. The 1 × 1 pointwise convolutions mix all channel information together, extracting cross-channel features and compensating for the inability of 7 × 7 depthwise convolutions to capture cross-channel features. Thus, the ConvNeXt block significantly reduces the number of parameters and computational cost while maintaining powerful feature extraction capabilities, enhancing the model’s computational efficiency. Moreover, the reduction in parameters also decreases the risk of model overfitting. Additionally, the use of layer normalization (layer normalization) and Gaussian error linear unit (GELU) activation functions within the ConvNeXt block further enhances the model’s training stability and nonlinear representation capabilities. Layer normalization helps stabilize the training process by normalizing the activations of each hidden layer, especially beneficial when training deeper networks. The GELU activation function provides a smooth nonlinearity, aiding in capturing complex patterns and relationships. The ConvNeXt block also includes residual connections, crucial for mitigating the vanishing gradient problem in deep networks. These connections allow gradients to flow directly through the network, enabling effective information propagation even in very deep networks, allowing the model to learn and express deeper features. In summary, the ConvNeXt block provides the model with a richer and more discriminative feature representation, crucial for improving the model’s performance in complex semantic segmentation tasks.

The UConvNeXt model begins with a standard 7 × 7 convolution, offering a large receptive field to capture extensive contextual information. Then, by stacking the ConvNeXt block, the encoder’s feature extraction capability is enhanced, meaning the decoder can utilize richer features for precise pixel-level prediction. Through its hierarchical structure design, the ConvNeXt model effectively captures features at different levels, from fine-grained textures to higher-level semantic features. This rich feature expression improves the model’s ability to recognize various patterns in images, especially in processing high-resolution images, capturing more refined details.

Overall, using ConvNeXt as the backbone provides U-Net with a powerful feature extraction mechanism, enhancing the model’s expressive and generalization abilities while improving parameter and computational efficiency. These improvements ultimately translate into performance gains in semantic segmentation tasks.

### Proposed AFOA-APOM algorithm

Yang et al*.* [28] introduced the aptenodytes forsteri optimization algorithm (AFOA), inspired by the movement strategies of aptenodytes forsteri during their warmth-seeking behavior. AFOA incorporates the concept of gradients to enhance the algorithm’s development capability and has achieved notable results in engineering optimization problems, combinatorial optimization algorithms, and scheduling issues. This research focuses on addressing the limitations of AFOA and, in combination with the characteristics of the deep learning semantic segmentation framework, proposes the adaptive perturbation of oscillation and mutation (APOM) operation. The APOM operation can be specifically divided into oscillation adaptive perturbation movement strategy I, simplified movement strategy II, global optimization movement strategy III, an adaptive selection strategy, and a mutation operation.

#### Oscillation adaptive perturbation movement strategy I

In the standard AFOA’s movement strategy I, the introduction of gradient theory for updating the optimal individual enhances the algorithm’s optimization capability. However, it is well known that in the deep learning framework, gradient methods have drawbacks such as a tendency to fall into local optima, limited applicability, and difficulty in determining an appropriate learning rate. Additionally, the computation of gradient values dimension-wise incurs high computational costs. Considering these issues, the APOM introduces oscillation adaptive perturbation movement strategy I to replace gradient computations. The individual update method for the maximization problem is as follows:

$$\begin{array}{c} X_{b}\left(t\right)=\\ \left\{\begin{array}{c} X_{b}\left(t-1\right)+v+\beta \;\text{if}\;f\left(X_{b}\left(t-1\right)+v+\beta \right)>f\left(X_{b}\left(t-1\right)-v-\beta \right)\\ X_{b}\left(t-1\right)-v-\beta \;\text{if}\;f\left(X_{b}\left(t-1\right)+v+\beta \right)<f\left(X_{b}\left(t-1\right)-v-\beta \right) \end{array}\right. \end{array}$$ (1)

$$v=\frac{{\left\|\lambda \right\|}^{0.8}*\;\lambda }{4^{*}\|\left(\mathrm{Ub}-\mathrm{Lb}\right)\|}$$ (2)

$$\begin{array}{c} \lambda =\text{exp}\left(\frac{-\;0.25^{*}{\mathrm{Ub}}^{*}\left(t-0.3^{*}\text{Maxg}\right)}{\text{Maxg}}\right)⊗\mathrm{rand}(1,\;D) \end{array}$$ (3)

$$\beta ={\mathrm{Ub}}^{*}q^{*}\lambda$$ (4)

where *X_b_*(*t*) represents the optimal individual, in which *t* denotes the current iteration number. *q* is either 0 or 1, with the probability of being 0 set at 0.5. *Ub* signifies the upper bound of the variable, while *Lb* indicates the lower bound. *Maxg* is the maximum runtime of the algorithm. *D* represents the dimensionality of the variable, β is a perturbation term, and λ is an adaptive change amount.

The improved movement strategy I, in the early stages of iteration, allows for a wider range of search around the optimal solution of the algorithm, which is beneficial in reducing the probability of the algorithm falling into premature convergence. In the later stages, it enables a narrower range of search around the optimal solution, which helps in minimizing the chances of missing the global optimal solution. Therefore, the improved movement strategy I exhibits excellent local search capabilities and the ability to escape local optima.

#### Simplified movement strategy II

In the standard AFOA’s movement strategy II, the standard deviation of the best position in each dimension from every aptenodytes’ memory is used to determine if the population is clustering prematurely. However, this approach has several issues: (a) The gradient value is computed for each dimension, resulting in a large computational load and high algorithmic complexity; (b) a small standard deviation in a single dimension does not necessarily mean that the individual distances are not large; (c) moving aptenodytes over shorter distances does not reduce the likelihood of individuals falling into local optima; (d) The method of determining premature clustering is not rational. Overall, the method in AFOA for determining premature clustering is not only irrational but also adds to the complexity of the algorithm, significantly slowing down the computational speed. To address these issues and reduce the complexity of the algorithm, AFOA-APM proposes a simplified movement strategy II, with the updated formula presented as Eq. 5.

$$\begin{array}{c} X_{i}\left(t\right)=\\ \left\{\begin{array}{c} X_{b}\left(t\right)+a^{*}\mathrm{rand}(1,\;D)⊗\left({\mathrm{Xm}}_{r1}\left(t\right)-{\mathrm{Xm}}_{r2}\left(t\right)\right)\;\text{if}\;{\mathrm{Xm}}_{r1}\left(t\right)\neq {\mathrm{Xm}}_{r2}\left(t\right)\\ X_{b}\left(t\right)+0.25^{*}\mathrm{rand}(1,\;D)⊗\left(\mathrm{Ub}-\mathrm{Lb}\right)\;\text{if}\;{\mathrm{Xm}}_{r1}\left(t\right)={\mathrm{Xm}}_{r2}\left(t\right) \end{array}\right. \end{array}$$ (5)

$$a=\text{exp}{\left(\frac{-d}{2^{*}D}\right)}^{0.8}$$ (6)

where *Ub* denotes the upper bound of the variable and *Lb* represents the lower bound. *Xm*_*r*1_(*t*) and *Xm*_*r*2_(*t*) are the optimal positions in the memory of two randomly selected aptenodytes forsteri. The term *d* from *a* represents the distance between *Xm*_*r*1_(*t*) and *Xm*_*r*2_(*t*).

Compared to the original AFOA, Eq. 5 eliminates the determination of premature convergence and updates according to individual dimensions, thereby reducing the complexity of the algorithm. When *Xm*_*r*1_(*t*) ≠ *Xm*_*r*2_(*t*), individuals in the population search in random directions near the optimal solution, enhancing the algorithm’s ability to escape local optima. Conversely, when *Xm*_*r*1_(*t*) = *Xm*_*r*2_(*t*), individuals will search toward potentially promising spaces, thus enhancing the algorithm’s local search capabilities.

#### Global optimization movement strategy III

In the standard AFOA’s movement strategy III, the update mechanism includes guidance from the optimal individual to other individuals, providing a balanced capability between local and global searches. However, an analysis of movement strategies I and II in the standard AFOA reveals that the former only updates the optimal individual, while the latter uses the globally optimal individual to update others in the population, both predominantly emphasizing local search capabilities. To enhance the global search capability in the early stages of the algorithm’s iterations, movement strategy III has been modified. Instead of guiding individuals toward the position of the optimal individual, it now guides them toward the position of a randomly chosen individual. This change allows aptenodytes forsteri to move randomly, greatly expanding the search range of the algorithm and strengthening its global search capability. The updated formula is presented as Eq. 7.

$$X_{i}\left(t\right)=X_{i}\left(t-1\right)+A_{i}\left(t\right)$$ (7)

$$\begin{array}{c} A_{i}\left(t\right)=\mathrm{rand}{\left(1,D\right)}^{*}A_{i}\left(t-1\right)+{a_{1}}^{*}\mathrm{rand}\left(1,D\right)⊗\\ \left(X_{r}\left(t\right)-X_{i}\left(t-1\right)\right)+{a_{2}}^{*}\mathrm{rand}\left(1,D\right)⊗\left({\mathrm{Xm}}_{i}\left(t\right)-X_{i}\left(t-1\right)\right) \end{array}$$ (8)

where *X_r_*(*t*) represents an individual randomly selected from the population. The parameters *a*_1_ and *a*_2_ are calculated using Eq. 6. In *a*_1_, the term *d* denotes the distance between *X_r_*(*t*) and *X_i_*(*t*), while in *a*_2_, *d* represents the distance between *Xm_i_*(*t*) and *X_i_*(*t*), and *D* indicates the dimensionality of the variables.

From Eq. 8, it is evident that the improved movement strategy III incorporates guidance toward the positions of random individuals. This change allows the individuals within the population to search in random directions within the solution space, thereby expanding the search range of the algorithm. As a result, this enhancement significantly strengthens the global search capabilities of the algorithm.

#### Mutation operation

For optimization problems, when the algorithm discovers a region with an extremum, individuals in the population will continuously converge toward this extremum point. As the number of iterations increases, the quantity of identical or similar individuals in the population gradually grows. In extreme cases, it is possible for all individuals in the population to become identical. Having a large number of identical or similar individuals can lead to a stagnation issue where the algorithm’s update operation fails to produce offspring that are different from their parents, thereby affecting the algorithm’s efficiency. To enhance the diversity of the algorithm while maintaining its inheritance properties, this study introduces a random mutation operation into the AFOA. The mutation operator can increase population diversity and reduce the likelihood of premature convergence. The steps for the random mutation operation are as follows: (a) randomly generate a 0 to 1 array with the probabilities of 0 and 1 set at 0.4 and 0.6, respectively; (b) randomly initialize the dimensions corresponding to 1.

Figure S3 illustrates the specific process of the random mutation operator. As can be seen from Fig. S3, after the random mutation operation, *X_i_*(*t*) not only inherits some characteristics of *X_i_*(*t* − 1) but also develops new features. The low similarity between *X_i_*(*t*) and *X_i_*(*t* − 1) indicates that the random mutation operation contributes not only to enhancing population diversity but also to maintaining the algorithm’s heredity.

#### Adaptive selection strategy

The performance of an algorithm is determined by its local and global search capabilities. Throughout the iterative process, global and local searches should alternate. After each global search, multiple local searches should be conducted. This approach effectively balances the global and local search capabilities of the algorithm, avoiding the trap of local optima while enhancing the algorithm’s convergence speed and solution accuracy. In the improved algorithm, the three types of movement strategies and the random mutation operation are designed to enhance these capabilities. Oscillatory adaptive disturbance movement strategy I and simplified movement strategy II exhibit stronger local search capabilities, whereas global optimization movement strategy III demonstrates enhanced global search capabilities. Additionally, the added random mutation operation increases population diversity. Therefore, to balance the global and local search capabilities of the algorithm, an adaptive selection probability *Pr* is employed to choose among the four updating strategies. The calculation formula for *Pr* is shown in Eq. 9.

$$\mathrm{Pr}=0.3+{\left(\frac{\mathrm{ti}}{2T_{\max}}\right)}^{g}$$ (9)

where *ti* represents the current iteration number, *T*_max_ denotes the maximum number of iterations for the algorithm, and *g* is the exponent.

The pseudocode for the adaptive selection strategy is shown in Algorithm 1.


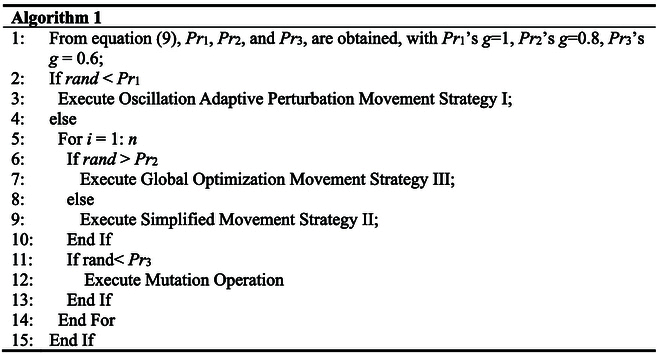


As can be seen from Algorithm 1, *Pr*_1_, *Pr*_2_, and *Pr*_3_ change incrementally from smaller to larger values. In the early stages of iteration, there is a higher probability of executing simplified movement strategy II, which endows the algorithm with strong exploratory capabilities. In the later stages, there is a higher probability of executing oscillation adaptive perturbation movement strategy I, global optimization movement strategy III, and mutation operation, enhancing the algorithm’s exploitation capabilities and aiding in maintaining the diversity of the population. Therefore, the adaptive changes in *Pr*_1_, *Pr*_2_, and *Pr*_3_ effectively balance the algorithm’s exploration and exploitation abilities, contributing to improved algorithmic performance.

### Multi-objective AFOA-APOM-based UConvNeXt structure optimization algorithm

#### Encoding process optimization

The structure optimization of the UConvNeXt model based on the multi-objective AFOA-APOM uses real number encoding and randomly generates decision variables within the range of [−0.5, 0.5]. Each decision variable corresponds to the change in the number of channels in each convolutional layer of the model. Assuming the population size is *n*, the number of convolutional layers in the UConvNeXt model is *m* and the number of channels in the convolutional layers is *L*, with the initial population being *X*. Therefore, *L* = (*l*_1_, *l*_2_, …, *l_v_*, …, *l_m_*), where *l_v_* represents the number of channels in the *v*th convolutional layer of the model; *X* = (*X*_1_, *X*_2_, …, *X_i_*, …, *X_n_*), where *X_i_* = (*x*^1^*_i_*, *x*^2^*_i_*, …, *x^*v*^*_i_*,* …, *x^*m*^*_i_**). Here, *X_i_* is an individual in AFOA-APM, and the *i*th windividual *X*i** in the initial population can be randomly i nitialized as follows:

$$X_{i}=\mathrm{rand}(1,\;m)-0.5$$ (10)

$$l_{v}^{\prime }=\left\lfloor l_{v}-{l_{v}}^{*}x_{i}^{v}\right\rfloor$$ (11)

where *rand*(1, *m*) is an *m*-dimensional random vector within the range of [0, 1]. x^*v*^*_i_* represents the *v*th component in the *i*th individual of the population, and *l*^*′*^*_v_* denotes the number of channels in the *v*th convolutional layer of the model after adjustment and ⌊⌋ indicates round down.

**Fig. 4.**
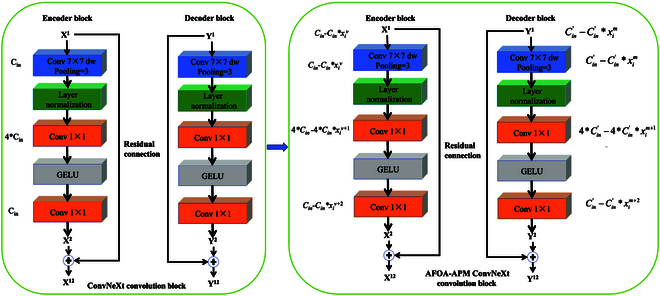
Schematic diagram of ConvNeXt convolution block adjusted by multi-objective AFOA-APM.

To illustrate the adjustment of the channels in the convolutional layers of the model, Fig. 4 presents the ConvNeXt convolution block after adjustment using the multi-objective AFOA-APM. Here, *C*_in_ represents the number of channels in the *v*th convolutional layer, x^*v*^*_i_* indicates the *v*th component in the *i*th individual within the AFOA-APM population, and x*_i_*^*v + 2*^ represents the (*v* + 2)th component in the *i*th individual of the population.

#### Establishment of multi-objective fitness function

The fitness function, used to evaluate the quality of solutions, is a crucial component in intelligent optimization algorithms. In this study, the speed and accuracy of identifying lodging crop areas are equally important. Therefore, when using AFOA-APM to automatically search for the optimal number of channels in convolutional layers of the UConvNeXt model, a balance should be struck between model performance and model complexity (in terms of the number of parameters and computational load). However, the objectives of model performance and complexity often conflict. Generally, increasing the complexity of the model can improve prediction accuracy, but it also increases the number of parameters and computational requirements, slowing down inference speed. Hence, a multi-objective fitness function is needed to balance these objectives and find a model that performs well across all targets.

Considering all these factors, the multi-objective fitness function for optimizing the number of channels in the convolutional layers of the UConvNeXt model using AFOA-APM is formulated as shown in Eq. 12.

$${\mathrm{f it}}_{i}=0.6^{*}{\text{mIoU}}_{i}+0.1^{*}P_{i}+0.3^{*}f_{i}$$ (12)

$$P_{i}=1-\frac{{\mathrm{pr}}_{i}}{\sum_{i=1}^{n}{\mathrm{pr}}_{i}}$$ (13)

$$f_{i}=1-\frac{{\mathrm{fl}}_{i}}{\sum_{i=1}^{n}{\mathrm{fl}}_{i}}$$ (14)

where *n* represents the size of individuals in the population, *pr_i_* is the number of parameters of the model corresponding to the *i*th individual, *fl_i_* is the computational load of the model corresponding to the *i*th individual, *P_i_* is the value related to the number of parameters of the *i*th individual, and *f_i_* is the value related to the computational load of the *i*th individual. mIoU*_i_* is the segmentation performance of the model corresponding to the *i*th individual on the rice lodging dataset.

### Loss function

Due to the randomness of rice lodging occurrences, the lodging category represents a lower proportion in the entire dataset. Even after data augmentation, a certain level of imbalance still exists among the four types of pixel classifications in the dataset. To further reduce the impact of this imbalance on the model, the UConvNeXt employs a combination of Focal_loss and Dice_loss as the loss function for rice lodging semantic segmentation tasks. This combination enhances the model’s predictive ability. Focal_loss was initially applied in object segmentation tasks to balance the loss between easy and difficult samples. It enables better optimization of parameters during backpropagation [29]. Dice_loss, on the other hand, is a region-based loss function that measures the overlap between the predicted segmentation area and the actual segmentation area [30]. Unlike pixel-level loss functions (such as cross-entropy loss), Dice_loss focuses more on the overall quality of segmentation, thereby improving segmentation accuracy. The combination of these two loss functions allows the model to consider both the global issue of class imbalance and the local quality of segmentation, increasing the model’s focus on difficult samples and enhancing its robustness for better segmentation results. The formulas for Focal_loss and Dice_loss are presented in Eqs. 15 and 16.

$$\text{Focal}\_\text{loss}=-\;{\left(1-p_{t}\right)}^{\gamma }\text{log}\left(p_{t}\right)$$ (15)

where *p*_t_ represents the confidence level of the predicted class for a sample and γ is a tuning parameter, typically set to 2 by default.

$$\begin{array}{c} \text{Dice}\_\text{loss}=1-\frac{2\sum_{i=1}^{\mathrm{Num}}y_{i}y_{\mathrm{il}}}{\sum_{i=1}^{\mathrm{Num}}y_{i}+\sum_{i=1}^{\mathrm{Num}}y_{\mathrm{il}}} \end{array}$$ (16)

where *y_il_* and *y_i_* respectively denote the label value and the predicted value for pixel *i*, while *Num* is the total number of pixels in the image.

### Evaluation metrics

To validate the effectiveness of the proposed model for rice lodging area segmentation, we utilize pixel accuracy (PA), mean pixel accuracy (MPA), intersection over union (IoU), mean intersection over union (mIoU), and F1 score.

PA is the most intuitive evaluation metric. It calculates the proportion of correctly predicted pixels by the model. The higher this value, the more accurate the model’s ability to locate class information. The formula for calculating PA is shown in Eq. 17.

$$\mathrm{PA}=\frac{\mathrm{Pc}}{\mathrm{PT}}$$ (17)

where *Pc* represents the number of pixels correctly predicted by the model, while *PT* denotes the total number of pixels in the image.

MPA evaluates the overall locational accuracy of the model by aggregating and then averaging the PA for each class. This metric is fairer than PA as it is not affected by class imbalance. The formula for calculating MPA is shown in Eq. 18.

$$\mathrm{MPA}=\frac{\sum_{i=1}^{\mathrm{Nm}}{\mathrm{PA}}_{i}}{\mathrm{Nm}}$$ (18)

where *Nm* represents the number of categories in the data and *PA_i_* is the pixel accuracy for the *i*th category.

IoU reflects the consistency between the segmented image and the manually annotated image in terms of both class pixels and background pixels. It calculates the ratio of the overlap between the target and the prediction result. The value of IoU ranges from 0 to 1, with values closer to 1 indicating better model segmentation performance. The formula for calculating IoU is shown in Eq. 19.

$$\mathrm{IoU}=\frac{\mathrm{TP}}{\mathrm{TP}+\mathrm{FP}+\mathrm{FN}}$$ (19)

where *TP* (true positives) represents the number of pixels that are correctly predicted as positive cases, *FP* (false positives) denotes the number of pixels that are negative cases but predicted as positive, and *FN* (false negatives) indicates the number of pixels that are positive cases but predicted as negative.

mIoU is a comprehensive metric that evaluates the overall segmentation accuracy of a model and considers the balance between different categories. In semantic segmentation tasks, mIoU is often used as a key evaluation metric to compare performance differences between various models or training setups. The method for calculating mIoU is shown in Eq. 20.

$$\text{mIoU}=\frac{\sum_{i=1}^{\mathrm{Nm}}{\mathrm{IoU}}_{i}}{\mathrm{Nm}}$$ (20)

where *Nm* denotes the number of categories in the data and *IoU_i_* is the intersection over union for the *i*th category.

The F1 score is an integrated metric for evaluating model performance. It is the harmonic mean of precision (P) and recall (R), considering both the model’s precision and recall, offering robustness against class imbalance issues. The F1 score provides a more nuanced assessment of model performance, particularly useful in handling complex scenes and objects with rich details.

$$P=\frac{\mathrm{TP}}{\mathrm{TP}+\mathrm{FP}}$$ (21)

$$R=\frac{\mathrm{TP}}{\mathrm{TP}+\mathrm{FN}}$$ (22)

$$F1\;\text{score}=\frac{2\mathrm{PR}}{P+R}=\frac{2\mathrm{TP}}{2\mathrm{TP}+\mathrm{FP}+\mathrm{FN}}$$ (23)

Confusion matrices serve as an insightful tool for understanding how well a model performs across different categories, pinpointing areas of strength and identifying categories where the model may face challenges. To facilitate a clearer understanding of confusion matrices, we present a confusion matrix for a four-category problem in Table 1. In this table, *TP_X_* represents the number of instances that are actually *X* and correctly predicted as *X*, *FP_YX_* represents the number of instances that are actually *Y* but incorrectly predicted as *X*, and *FN_XY_* represents the number of instances that are actually *X* but incorrectly predicted as *Y*.

**Table 1.** The confusion matrix

True category/predict category	Predict category is *A*	Predict category is *B*	Predict category is *C*	Predict category is *D*
True category is *A*	*TPA*	*FN_AB_*	*FN_AC_*	*FN_AD_*
True category is *B*	*FP_BA_*	*TP_B_*	*FN_BC_*	*FN_BD_*
True category is *C*	*FP_CA_*	*FP_CB_*	*TP_C_*	*FN_CD_*
True category is *D*	*FP_DA_*	*FP_DB_*	*FP_DC_*	*TP_D_*

Beyond the standard confusion matrix, the normalized confusion matrix is also an important tool. It involves normalizing the values in each row by dividing them by the sum of values in that row so that the sum of each row equals 1. This normalization process helps us compare classification performance between different categories more intuitively because it reflects the distribution of predicted categories for a given true category as relative proportions. The normalized confusion matrix is particularly useful in dealing with class imbalance, as it provides a more balanced perspective on performance assessment by showing information in relative proportions rather than absolute numbers.

In the normalized confusion matrix, the diagonal elements represent the recall for each category; for each category *X*, its recall can be represented as *TP_X_* divided by the total number of true instances for that category (*TP_X_* plus all instances of *X* incorrectly predicted as other categories). Observing the diagonal of the normalized confusion matrix allows us to quickly assess the model’s accuracy in identifying each category. Meanwhile, the off-diagonal elements provide insights into the confusion scenarios of the model, showing the relative frequency at which instances of one category are incorrectly predicted as another, helping us identify potential difficulties the model may have in distinguishing between certain categories.

To validate the lightweight performance of the proposed model for rice lodging area segmentation, we utilize the parameters and floating-point operations per second (FLOPs) as the evaluation metrics. These metrics provide insight into the efficiency and computational resource requirements of the model, crucial for practical applications where computational resources might be limited.

### System setup

To ensure the fairness of results, all experiments are conducted on the same computer. To ascertain the segmentation accuracy and computational time of the proposed networks, the experiments are executed on a Windows 10 operating system. The computer is equipped with a Core i7-13700KF @ 3.40 GHz CPU and 64 GB RAM. Python is used as the programming language, with PyCharm as the compiler. Network model construction, training, and testing are conducted using the Pytorch deep learning framework. An NVIDIA GeForce RTX 4090 with 24 GB of memory is employed to facilitate network training during the experimentation process. The rice lodging dataset, described in the “Dataset” section, was employed to train all models, with the dataset being divided into a training set and a validation set at a ratio of 0.75:0.25. This division resulted in 2,956 images allocated to the training set and 535 images to the validation set. For the optimization of CNN structure parameters with the multi-objective AFOA-APOM, the population size is set to 8, and the algorithm iterates 10 times. All participating models undergo 500 epochs of training, with a batch size set to 4. The model optimizer is AdamW. The initial learning rate is set at 0.0015 with a weight decay coefficient of 5 × 10^−5^.

## Results and Discussion

### The structure of AAUConvNeXt

**Fig. 5.**
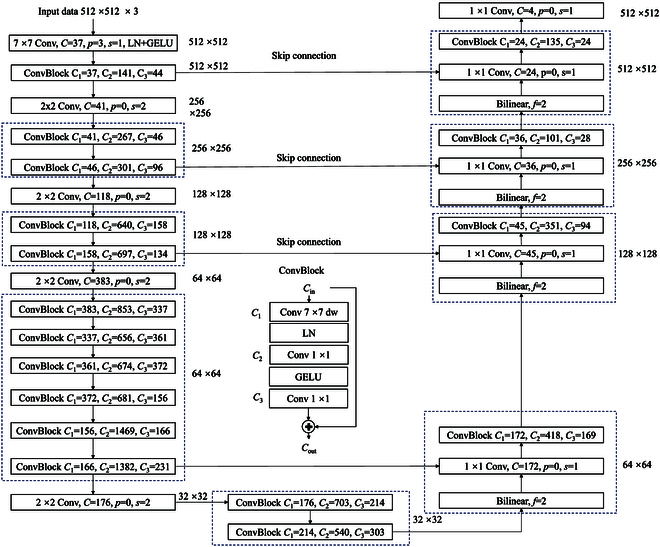
The structure of AAUConvNeXt model.

The structural diagram of the model is presented in Fig. 5, which represents the optimized version of the UConvNeXt model achieved through the multi-objective AFOA-APM optimization. In the diagram, *C*, *C*_1_, *C*_2_, and *C*_3_ denote the number of output channels for the convolutional layers, *p* denotes the number of padding units, *s* represents the stride of the convolutional kernel, Bilinear refers to bilinear interpolation used for resizing the feature maps, and *f* is the scale factor indicating the multiple by which the image size is increased during bilinear interpolation. A factor of *f* = 2 means that the image dimensions are doubled both horizontally and vertically.

As seen from Fig. 5, compared to the UConvNeXt model, the AAUConvNeXt model maintains the same positioning for convolutional and pooling layers; however, there is a significant change in the number of channels in the convolutional layers. In the UConvNeXt model, the number of channels in the convolutional layer doubles with the reduction of feature map sizes during the down-sampling process and halves with the increase in feature map sizes during the up-sampling process. Traditional models enhance the network’s learning capability by increasing the number of convolutional layer channels, but this also means higher computational costs and memory usage. After algorithm optimization, the number of channels in the AAUConvNeXt model’s convolutional layers no longer adheres to a simple principle of multiplicative increase or decrease. Through the optimization process of the algorithm, the model increases the number of channels in key layers that significantly contribute to learning features of rice lodging data for the task of segmenting lodging rice areas while reducing the number of channels in noncritical layers to balance model complexity and performance. By precisely adjusting the number of channels through the algorithm, the model’s accuracy and generalization ability in segmenting lodging rice areas under limited computational resources are improved.

### Ablation experiment

To compare the performance of three different models, the UConvNeXt model, the UConvNeXt model optimized by AFOA, and the UConvNeXt model improved with AFOA-APOM were conducted, as shown in Table 2. In this context, AUConvNeXt represents AFOA combined with UConvNeXt, while AAUConvNeXt signifies the combination of AFOA, APOM, and UConvNeXt.

**Table 2.** Ablation experiment results

Evaluation metrics	UConvNeXt	AUConvNeXt	AAUConvNeXt
PA (%)	95.2	95.9	96.3
MPA (%)	95.2	96.0	96.3
mIoU (%)	91.3	92.5	93.2
Parameters (M)	5.83	5.23	4.87
FLOPs (G)	4.04	3.87	3.69

From Table 2, it is evident that with the addition of different strategies, the model’s performance improved across the PA, MPA, and mIoU metrics. Both AUConvNeXt and AAUConvNeXt achieved higher PA, MPA, and mIoU than UConvNeXt, with a decrease in the number of parameters and FLOPs by 4.21% and 8.66%, respectively. Compared to the baseline UConvNeXt model, the AUConvNeXt model optimized using AFOA improved PA, MPA, and mIoU by 0.7%, 0.8%, and 1.2%, respectively. The AAUConvNeXt model, employing the APOM optimization strategy, further improved PA, MPA, and mIoU by 1.1%, 1.1%, and 1.9%, respectively. When compared with the AUConvNeXt model, the AAUConvNeXt model showed increases of 0.4%, 0.3%, and 0.7% in PA, MPA, and mIoU, respectively. These results indicate that the utilization of APOM to optimize the traditional AFOA can significantly enhance the model’s performance based on the baseline [31], with improvements observed both in mIoU accuracy and in terms of parameter count and operational speed. This analysis suggests that the approach of using multi-objective AFOA-APOM to optimize the number of channels in the convolutional layers of a semantic segmentation model is feasible, and the two-stage training strategy is effective.

### Comparison of model channel numbers after multi-objective AFOA-APOM optimization

The number of channels in the convolutional layers of a CNN model greatly influences its complexity and performance. This observation is consistent with the findings of Yang et al*.* [32], who noted that while increasing the number of channels can expand the receptive field, it also increases computational time and training difficulty, and can decrease efficiency. Table 3 shows the reduction ratio of the number of channels in each convolutional layer of the model before and after optimization using the multi-objective AFOA-APOM algorithm. In this context, DSCL (down-sampling convolutional layer) refers to the convolutional layers in down-sampling, and USCL (up-sampling convolutional layer) refers to those in up-sampling [the convolutional layers in Table 3 do not include the depthwise convolutional (DW) layers in the model]. As can be seen from Table 3, the number of channels in 23 feature layer convolutional layers increases after optimization with the multi-objective AFOA-APOM algorithm, while the remaining 20 channels decrease. The results indicate that this optimization process does not simply increase or decrease the number of channels indiscriminately, but rather adjusts them in a targeted manner according to the model’s requirements and performance at different stages. In the initial stages (such as DSCL1-DSCL10 and USCL1-USCL6), there is a notable increase in the number of channels. This is likely because the model requires more features to capture and express the complexity of the input data in these early stages. This observation is consistent with the findings of Leite and Xiao [33], who discovered that increasing the number of channels in the early stages of feature extraction in deep learning models can effectively enhance the F1 score. This underscores the crucial role of the model’s early-stage feature recognition capabilities in determining overall performance. Additionally, Sunil et al*.* [34] have proposed that increasing the number of channels can aid the model in better learning and understanding the data. However, this strategy of increasing the number of channels may lead to increased model complexity. Therefore, in the later stages, the algorithm begins to seek ways to reduce the number of channels to optimize model complexity. In the later stages, such as DS11-DS31 and US7-US12, there is a more frequent reduction in the number of channels. This reduction is likely because, after learning complex features in the initial stages, the model has already acquired sufficient feature representation. Hence, in the later stages, it is possible to reduce model complexity by decreasing the number of channels while maintaining model performance. This approach is a key aspect of the current trend toward simplifying deep learning models. Liu et al*.* demonstrated that reducing the number of channels in deep CNNs through network slimming techniques can decrease the model size, reduce runtime memory usage, and lessen computational operations without compromising accuracy. This validates the effectiveness of reducing the number of channels in the later stages of the model to optimize complexity [35].

**Table 3.** Convolutional layer channel counts of the segmentation models before and after optimization

Convolutional layer	UConvNeXt	AAUConvNeXt	Reduction ratio	Convolutional layer	UConvNeXt	AAUConvNeXt	Reduction ratio
DSCL 01	32	37	−15.63%	DSCL 23	1,024	1,469	−43.46%
DSCL 02	128	141	−10.16%	DSCL 24	256	166	35.16%
DSCL 03	32	44	−37.50%	DSCL 25	1,024	1,382	−34.96%
DSCL 04	64	41	35.94%	DSCL 26	256	231	9.77%
DSCL 05	256	267	−4.30%	DSCL 27	256	176	31.25%
DSCL 06	64	46	28.13%	DSCL 28	1,024	703	31.35%
DSCL 07	256	301	−17.58%	DSCL 29	256	214	16.41%
DSCL 08	64	96	−50.00%	DSCL 30	1,024	540	47.27%
DSCL 09	128	118	7.81%	DSCL 31	256	303	−18.36%
DSCL 10	512	640	−25.00%	USCL 01	128	172	−34.38%
DSCL 11	128	158	−23.44%	USCL 02	512	418	18.36%
DSCL 12	512	697	−36.13%	USCL 03	128	169	−32.03%
DSCL 13	128	134	−4.69%	USCL 04	64	45	29.69%
DSCL 14	256	383	−49.61%	USCL 05	256	351	−37.11%
DSCL 15	1,024	853	16.70%	USCL 06	64	94	−46.88%
DSCL 16	256	337	−31.64%	USCL 07	32	36	−12.50%
DSCL 17	1,024	656	35.94%	USCL 08	128	101	21.09%
DSCL 18	256	361	−41.02%	USCL 09	32	28	12.50%
DSCL 19	1,024	674	34.18%	USCL 10	32	24	25.00%
DSCL 20	256	372	−45.31%	USCL 11	128	135	−5.47%
DSCL 21	1,024	681	33.50%	USCL 12	32	24	25.00%
DSCL 22	256	156	39.06%				

Moreover, it is noteworthy that in some layers (DSCL8, DSCL14, DSCL20, and USCL6), the increase in the number of channels is exceptionally high (exceeding 40%). This suggests that these layers may play a significant role in the model’s ability to learn data features, and a smaller number of channels might not sufficiently capture the important features of the input data. This observation aligns with the research of Wang et al. [36], which indicated that increasing the feature extraction channels in critical layers of deep learning network structures can effectively prevent the loss of key spatial information, thereby better learning deep features. Therefore, the optimization algorithm increases the number of channels in these layers, which may help enhance the model’s performance. In some layers, there is a high reduction ratio in the number of convolutional layer channels, indicating that these layers may have a lesser role in learning data features. Substantially reducing the number of channels in these layers can decrease the model’s complexity without significantly impacting its performance. Cheng et al*.* [37] have conducted in-depth research in this area, finding that reducing the number of channels in feature maps can decrease the number of parameters without significantly affecting the model’s performance. This finding is also consistent with our research results. It shows that by using intelligent optimization algorithms, different optimization strategies can be employed at various stages to effectively adjust the number of channels in the model, achieving a balance between model complexity and performance. This optimization strategy offers a potential solution for enhancing the computational efficiency and performance of the model.

### Model performance comparison

#### Accuracy and loss

**Fig. 6.**
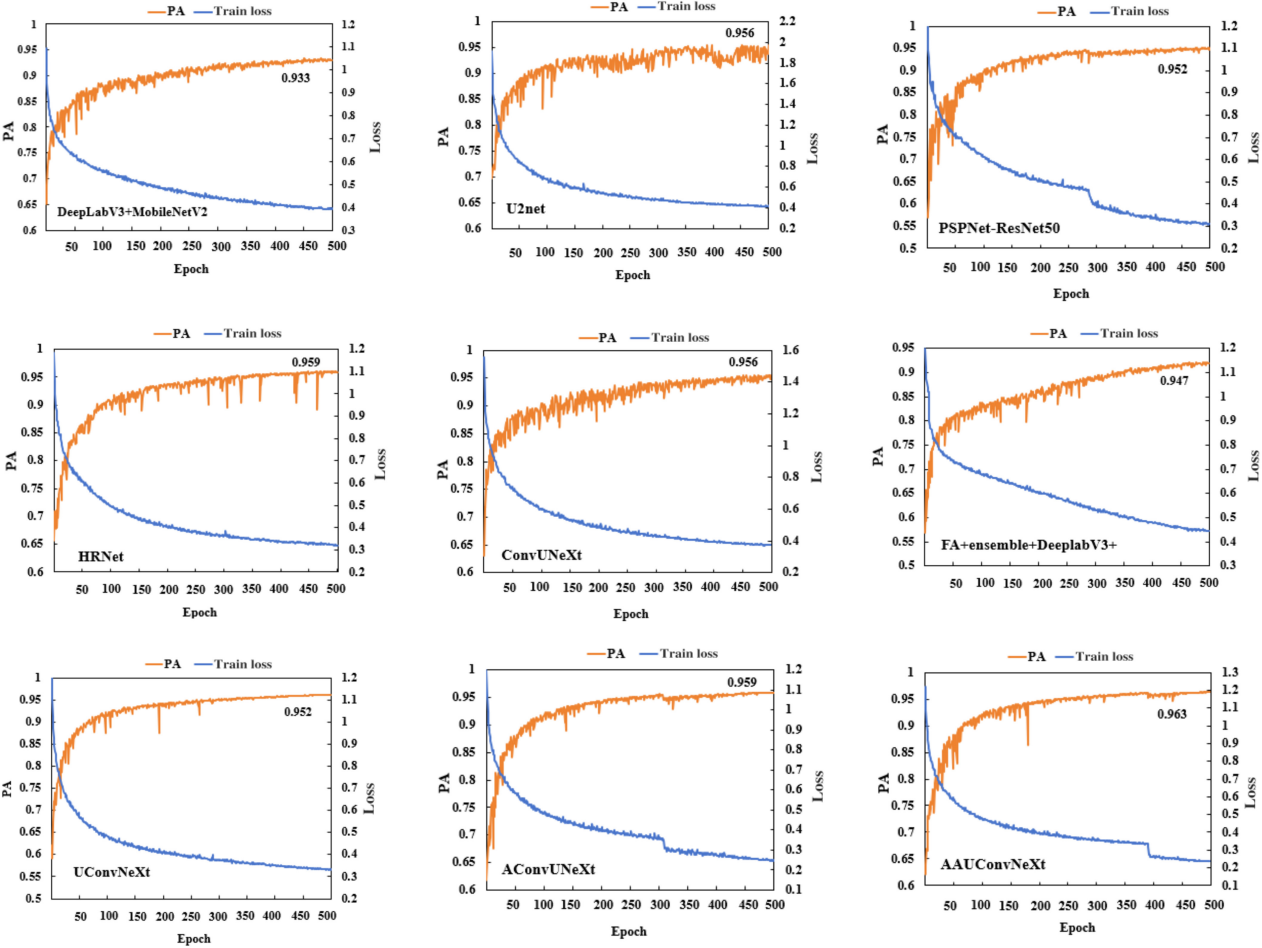
The PA and training loss function graph of the SOTA models for identifying rice lodging.

To demonstrate the performance differences between the proposed AAUConvNeXt and existing SOTA models, Fig. 6 illustrates the loss and accuracy variation curves during the training process of nine deep learning models: DeepLabV3+ with the backbone MobileNetV2 (DeepLabV3 + MobileNetV2) [38], U2Net [39], PSPNet with the backbone Resnet-50 (PSPNet-ResNet50) [40], HRNet [41], ConvUNext [42], FA + ensemble + DeepLabV3+ [43], UConvNeX, AUConvNeXt, and AAUConvNeXt. From the loss curve in Fig. 6, it can be observed that the loss trends of the nine models are fundamentally similar. At the beginning of training, the model loss decreases rapidly; the speed of loss reduction slows down in the middle phase and stabilizes in the later stages. After 500 training epochs, the loss of the AAUConvNeXt model converges around 0.23, followed by AUConvNeXt at 0.25, with HRNet and PSPNet-ResNet50 converging around 0.3. From the PA curve, it is apparent that for the UConvUNext, AUConvUNext, ConvUNext, DeepLabV3 + MobileNetV2, FA + ensemble + DeepLabV3+, HRNet, AAUConvNeXt, PSPNet-ResNet50, and U2Net models, their PA reached 0.952, 0.959, 0.956, 0.933, 0.947, 0.959, 0.963, 0.952, and 0.956, respectively. This indicates that after extensive iterative training, these deep learning models are capable of efficiently recognizing the characteristics of crop lodging, thereby enabling precise segmentation of crop lodging areas. Notably, the AAUConvNeXt model demonstrated superior performance, achieving a PA of 0.963, leading all compared models. This highlights the efficiency and superiority of our proposed model in handling the task of crop lodging segmentation. The improved performance of the AAUConvNeXt model can be attributed to the multi-objective adaptive algorithm used for optimization, which endows it with finer feature extraction capabilities and higher pixel classification accuracy in complex agricultural scenarios. The characteristics of the AAUConvNeXt model, such as its lower loss values and higher accuracy, clearly demonstrate its potential in the field of crop lodging segmentation research among deep learning models.

#### Segmentation results of semantic segmentation model

**Fig. 7.**
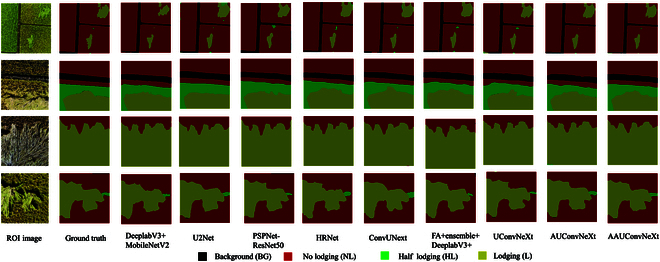
Segmentation effects of nine semantic segmentation models.

To visually validate the effectiveness of the proposed strategies, Fig. 7 presents the segmentation results of nine semantic segmentation models across four sets of rice images, showcasing their performance in segmenting areas of rice lodging at various degrees.

From Fig. 7, it can be observed that for the categories BG and NL, most methods exhibit good segmentation performance. This could be attributed to the distinct features of these two categories, which are less likely to be confused with other categories, and the abundance of samples. The quality of samples directly influences the model’s performance [44], Moreover, most studies on crop lodging focus only on the discussion of L or NL areas [45], with very limited research on partially lodging situations.

Compared with the segmentation effect of BG and NL categories, most methods show lower accuracy in identifying HL and L categories. This is due to the high similarity in features between the edges of the L category and the HL category in the rice lodging dataset. However, the AUConvNeXt and AAUConvNeXt models exhibit significantly higher accuracy in recognizing HL and L categories compared to other models. This is because these models, after being optimized through the multi-objective AFOA algorithm, effectively adjusted the number of channels in each convolutional layer of the model. This optimization strategy makes targeted adjustments based on the needs and performance of the model at different stages. By increasing the number of channels in certain critical feature layers, the model’s capability to express features is enhanced, enabling it to learn more complex features.

**Fig. 8.**
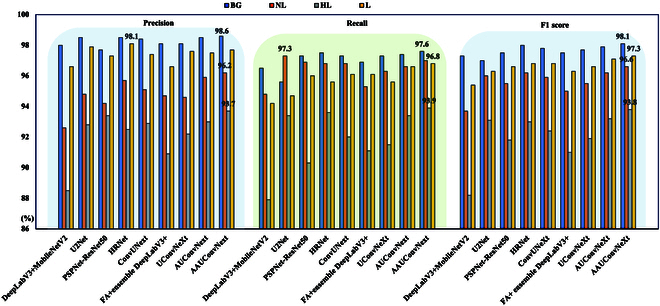
Precision, recall, and F1 score results of nine SOTA models.

Figure 8 presents the precision, recall, and F1 score metrics for each category of lodging rice data across the nine models. Figure 8 demonstrates the performance of various semantic segmentation models in segmenting rice lodging areas across different categories. In our analysis of the performance metrics across all models, we observed that for the NL category, both precision and F1 scores are consistently lower than those for the L category. However, in terms of recall, the NL category surpasses the L category in the majority of models. This discrepancy underscores the distinct insights that precision, recall, and F1 score metrics contribute to understanding model performance. A higher recall for the NL category suggests that the models miss fewer instances of this category, potentially due to the more distinct or widely distributed features of the NL category within the dataset, facilitating more accurate identification of NL states by the models. Conversely, the lower precision for the NL category compared to the L category might indicate that there are more false positives within the samples predicted as NL, meaning that some samples actually belonging to the HL or L categories might be incorrectly predicted as NL, thereby reducing the precision for NL. The F1 score, being the harmonic mean of precision and recall, aims to balance both metrics. The lower F1 score for the NL category relative to the L category suggests that despite the higher recall, the lower precision negatively impacts the F1 score, resulting in a lower overall performance evaluation for the NL category compared to the L category. This analysis highlights the complexity of evaluating model performance in semantic segmentation tasks and underscores the importance of considering multiple metrics to gain a comprehensive understanding of a model’s capabilities and limitations in distinguishing between different lodging states.

Among all the models, HRNet exhibits the highest precision for the L category. However, its precision for the NL category falls below that of AUConvNeXt and AAUConvNeXt, and in the HL category, it is surpassed by U2Net, PSPNet-ResNet50, ConvUNeXt, AUConvNeXt, and AAUConvNeXt. On the other hand, U2Net shows the best recall performance in the NL category but has lower recall than HRNet, AUConvNeXt, and AAUConvNeXt in the BG, HL, and L categories. This pattern may suggest a certain bias in HRNet toward the L category and in U2Net toward the NL category, indicating lower stability in these models’ performance across different categories. Both AUConvNeXt and AAUConvNeXt models demonstrate superior performance across precision, recall, and F1 score metrics, particularly notable in F1 scores. Except for the BG category, where AUConvNeXt performs slightly lower than HRNet, and the NL category, where it equals HRNet, AUConvNeXt and AAUConvNeXt exceed the performance of other models in the remaining categories. Moreover, AAUConvNeXt stands out as the best-performing model among the nine models, with the exception of having a lower precision in the L category compared to HRNet and a lower recall in the NL category compared to U2Net. The remaining metrics significantly outperform the other models; this indicates a better stability of AUConvNeXt and AAUConvNeXt.

#### Confusion matrices

**Fig. 9.**
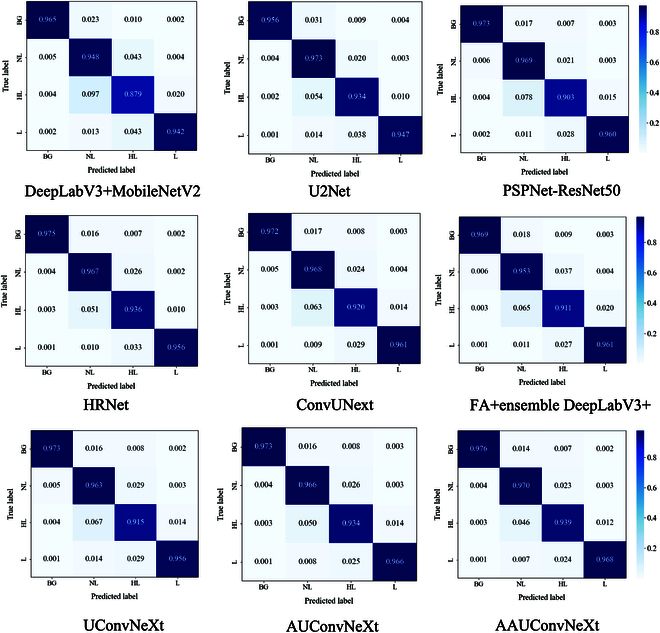
Confusion matrices pertaining to rice lodging segmentation.

By comparing the performance of models under different metrics, their stability can be assessed. A good model should not only perform well under all conditions but also show relatively consistent performance under different conditions. As can be seen from the above PA, MPA, mIoU, F1 score, recall, precision, and segmentation effects, both AUConvNeXt and AAUConvNeXt demonstrate superior performance, indicating high stability of the models. Additionally, to present the segmentation performance of each model more clearly across various categories, Fig. 9 provides the confusion matrices for the nine models. In Fig. 9, each row of the matrix has been normalized to facilitate an intuitive comparison across categories.

From the confusion matrices, it can be observed that AAUConvNeXt performs better than the eight comparative algorithms from the literature in all categories except for the NL category, where it is slightly lower than U2Net. This indicates the effectiveness of using the multi-objective AFOA-APM to optimize the channels in the model’s convolutional layers. The segmentation accuracy of AUConvNeXt in the L category, excluding AAConvNeXt, stands out as the highest among the models evaluated, while HRNet achieves the highest segmentation accuracy in the HL and BG categories. This performance underscores the robust capabilities of both models. The effectiveness of the multi-objective AFOA in optimizing the channels of the model’s convolutional layers is particularly evident in AUConvNeXt’s performance. Such optimization allows for a more efficient and targeted feature extraction process, contributing significantly to the model’s precision in specific categories. HRNet distinguishes itself by maintaining high-resolution feature maps throughout the entire network, a departure from traditional deep learning models that typically reduce resolution in the early stages of the network. By preserving high resolution, HRNet possesses enhanced feature representation capabilities, enabling the capture of fine-grained information. Consequently, HRNet can maintain higher segmentation accuracy in certain categories, illustrating the advantages of its architecture in handling complex segmentation tasks.

Moreover, all models show lower segmentation accuracy for the HL category compared to the other three categories. This is due to the similar features of NL and HL categories, and the presence of features at the edges of the L category that resemble those of HL, resulting in the lowest segmentation accuracy for the HL category among the nine models. Among all comparative methods, DeepLabV3 + MobileNetV2 shows the lowest category segmentation accuracy, possibly due to its fusion strategy’s ineffectiveness in handling information with a high degree of similarity. This finding diverges to some extent from the conclusions of Zhang et al*.* [46], potentially due to DeepLabV3 + MobileNetV2’s weaker capability in capturing detailed texture features of crops in specific scenarios. The DeepLabV3 + MobileNetV2 model employs MobileNetV2 as its backbone network, which is initially designed to balance accuracy and efficiency. Consequently, its lightweight structure may exhibit limitations in capturing complex textures and fine details. This limitation becomes particularly evident when processing rice lodging scenes with highly similar information. Moreover, the rice lodging dataset features highly diverse environmental conditions, complex background textures, and varying degrees of lodging. These characteristics necessitate a model with a larger receptive field and higher model capacity to effectively capture and distinguish various texture features. The relatively small receptive field and limited model capacity of MobileNetV2 might not meet these requirements, leading to deficiencies in classification accuracy.

#### The overall performance of the SOTA models

Table 4 shows that among the nine models, AAUConvNeXt excels in performance, achieving the highest PA at 96.3%, MPA at 96.3%, and mIoU at 93.2%. This indicates its outstanding accuracy and effectiveness in image semantic segmentation tasks. AUConvNeXt also performs admirably, with a high PA of 95.9%, MPA of 96.0%, and mIoU of 92.5%, striking a good balance between performance and resource efficiency. The HRNet model also achieves high performance, especially in mIoU at 92.4%, but it has a relatively higher parameter count and computational complexity. AUConvNeXt and AAUConvNeXt models show a clear advantage in terms of parameter count and computational complexity among the nine models. In terms of computational demand, apart from ConvUNext and DeepLabV3 + MobileNetV2 models, AUConvNeXt and AAUConvNeXt have lower computational complexity relative to the other comparative models. The parameter of AAUConvNeXt is only higher than the ConvUNext model among the six comparative models, suggesting the effectiveness in situations with limited computational resources.

**Table 4.** Performance of different models in locating rice lodging areas

Model	Parameters (M)	FLOPs (G)	PA (%)	MPA (%)	mIoU (%)
DeepLabV3 + MobileNetV2	5.81	2.64	93.3	93.3	88.2
U2Net	44.04	15.13	95.6	95.2	91.7
PSPNet-ResNet50	46.7	5.92	95.2	95.1	91.2
HRNet	65.85	9.39	95.9	95.9	92.4
ConvUNext	3.50	2.87	95.6	95.5	91.9
FA + ensemble + DeepLabV3+	67.35	4.78	94.7	94.6	90.4
UConvNeXt	5.83	4.04	95.2	95.2	91.3
AUConvNeXt	5.23	3.87	95.9	96.0	92.5
AAUConvNeXt	4.87	3.69	96.3	96.3	93.2

## Conclusion

To effectively address the challenges of rice lodging segmentation, this study introduces a novel approach using the AFOA + APOM + UConvNeXt network architecture. This method utilizes ConvNeXt as its backbone, incorporating AFOA-APOM intelligent optimization algorithms to improve the model’s performance. Specifically designed for the intricacies of crop lodging segmentation, such as adaptive optimization of convolutional layer channels, the model significantly increases the accuracy of segmenting rice lodging areas, reduces the model’s parameter count, and enhances computational speed, effectively lowering the consumption of computational resources. Compared to eight SOTA models, AAUConvNeXt excels in performance, achieving the highest PA of 96.3%, MPA of 96.3%, and mIoU of 93.2%. This demonstrates its exceptional accuracy and efficacy in image semantic segmentation tasks. Additionally, AAUConvNeXt’s parameter and computational complexity are notably advantageous among the nine models considered. Overall, AAUConvNeXt maintains high segmentation accuracy for lodging, HL, and NL areas. The integration of deep learning with intelligent optimization algorithms in this study represents a significant advancement in the field of crop phenotypic information extraction. Future research is aimed at exploring the segmentation of HL areas, which is expected to aid in making precise predictions of lodging trends, facilitating the acquisition of rice phenotypic information, and promptly addressing yield losses due to crop lodging.

## Data Availability

The data used to support the findings of this study are available from the corresponding author upon request.
