# Supplementary figures and images for: AAUConvNeXt: Enhancing Crop Lodging Segmentation with Optimized Deep Learning Architectures
Source: Plant Phenomics. 2024 Apr 25;6:0182. doi: 10.34133/plantphenomics.0182 (PMC11654911; doi:10.34133/plantphenomics.0182)

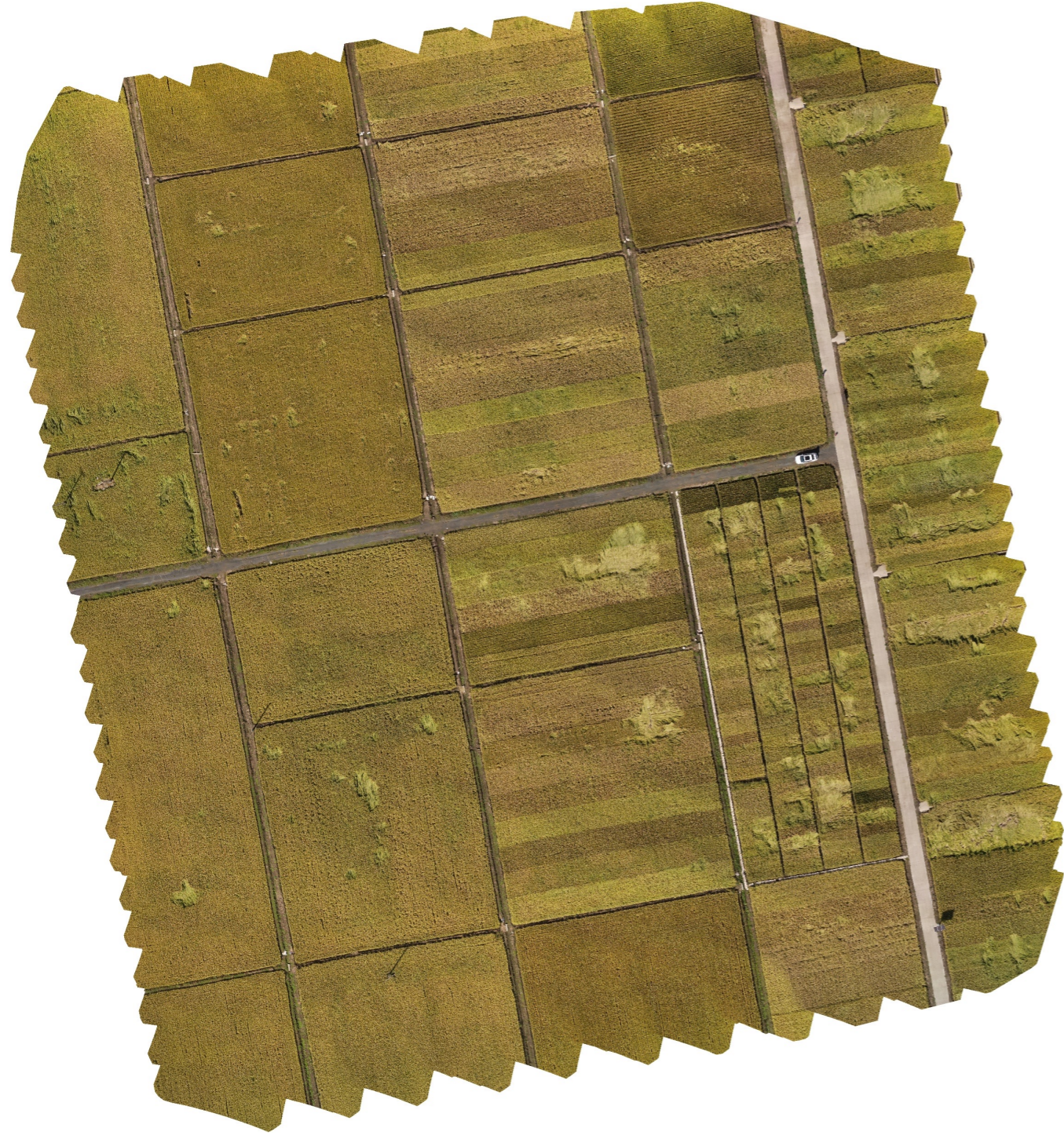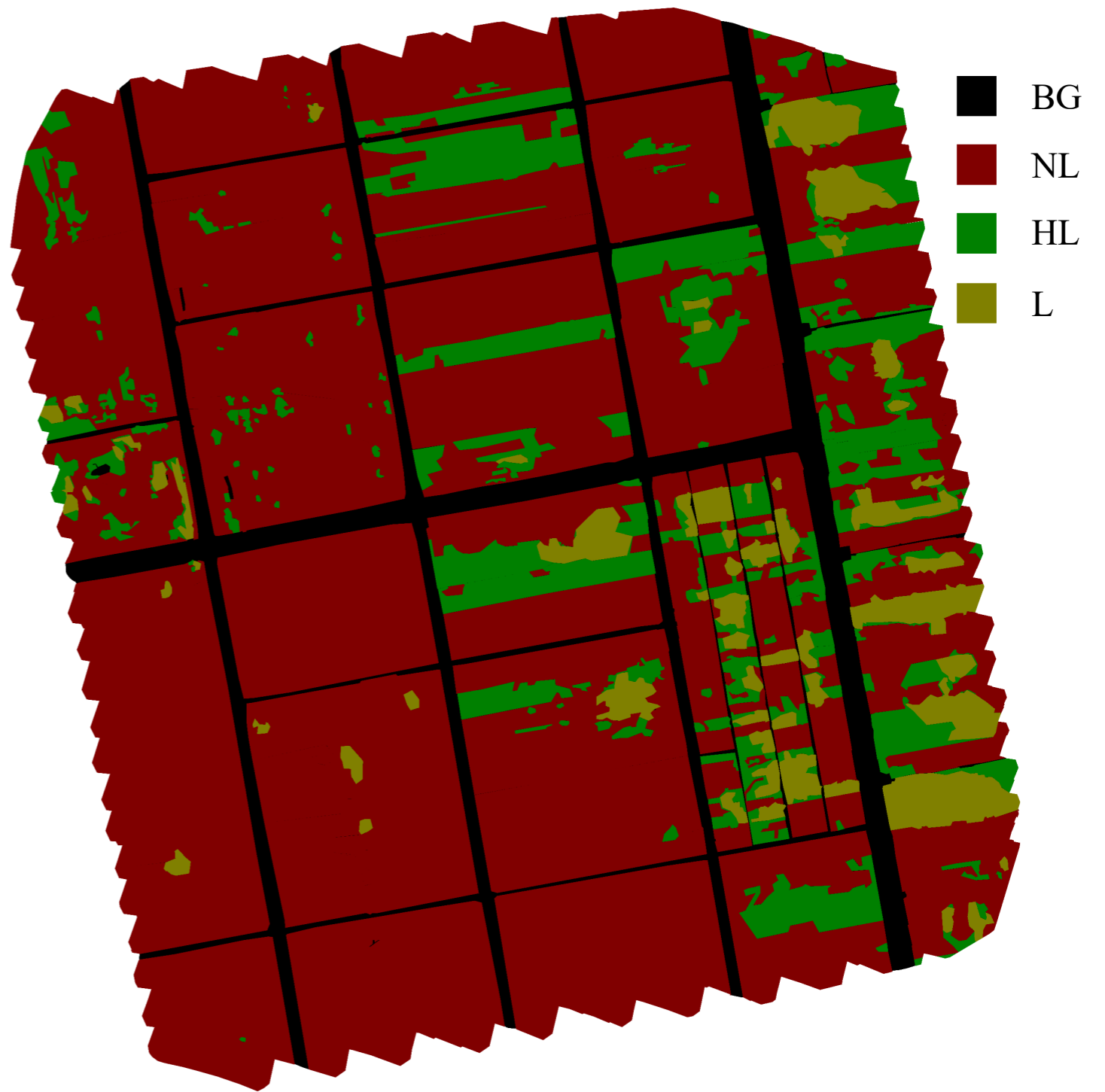

Supplement: Supplementary 1 — Figs. S1 to S3 [file plantphenomics.0182.f1.zip › Fig.1S.pdf]

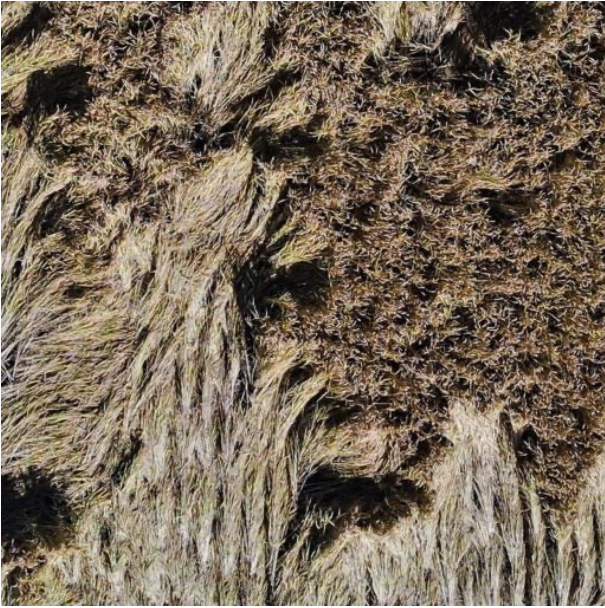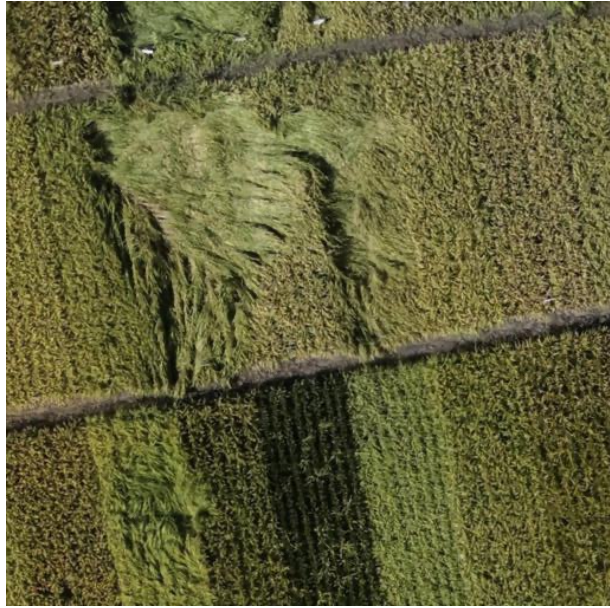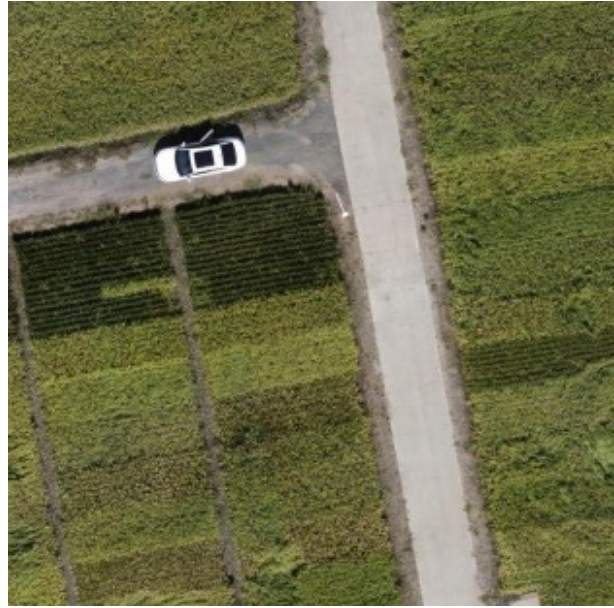

Supplement: Supplementary 1 — Figs. S1 to S3 [file plantphenomics.0182.f1.zip › Fig.2S.pdf]

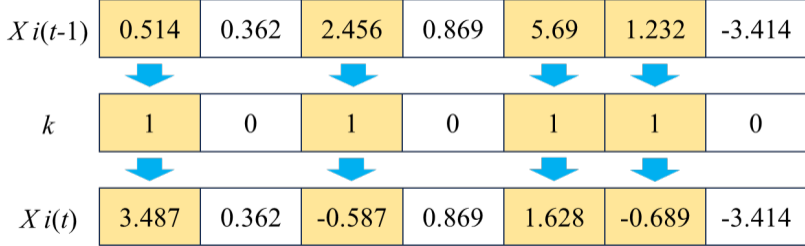

Supplement: Supplementary 1 — Figs. S1 to S3 [file plantphenomics.0182.f1.zip › Fig.3S.pdf]
